# Household- and school-level parental education and academic self-concept development in elementary school

**DOI:** 10.1038/s41539-025-00354-x

**Published:** 2025-08-26

**Authors:** Nil Horoz, Nienke van Atteveldt, Joost Oude Groeniger, Tanja A. J. Houweling, Frank J. van Lenthe, TuongVan Vu, Hans M. Koot, J. Marieke Buil

**Affiliations:** 1 Department of Clinical, Neuro- and Developmental Psychology, Vrije Universiteit Amsterdam, Amsterdam, The Netherlands; 2 Amsterdam Public Health Research Institute, Amsterdam, The Netherlands; 3 Research Institute Learn!, Vrije Universiteit Amsterdam, Amsterdam, The Netherlands; 4 Department of Public Health, Erasmus MC, University Medical Center Rotterdam, Rotterdam, The Netherlands; 5 Department of Public Administration and Sociology, Erasmus University Rotterdam, Rotterdam, The Netherlands; 6 Department of Human Geography and Spatial Planning, Faculty of Geosciences, Utrecht University, Utrecht, The Netherlands

**Keywords:** Education, Psychology

## Abstract

This longitudinal study examined the main effect associations and cross-level interactions of household- and school-level parental education on academic self-concept (ASC) development from fourth to sixth grade of elementary school. Furthermore, the mediating roles of child- and school-level academic achievement (AA) in these associations were examined. Children (*N* = 679, ages 10–12) from 18 elementary schools were followed annually. ASC levels were relatively high and stable from fourth to sixth grade. Results showed that lower household-level parental education was indirectly associated with lower child-level ASC through lower child-level AA. Lower school-level AA and tentatively higher school-level ASC scores were found in lower parental education schools compared to higher parental education schools. School-level AA was not associated with school-level ASC. Furthermore, results showed initial support that, in terms of ASC, children of lower-educated parents may benefit slightly more from attending lower parental education schools than attending higher parental education schools.

## Introduction

The elementary school period is a window for the promotion of equal opportunities for all children to reach their full potential and to reduce inequalities in educational outcomes^1,2^. Extant research shows that differences in parental education at both the household and school levels contribute to disparities in children’s overall development and their educational outcomes. These contributions are visible across the elementary and secondary school years^1,3–8^. Moreover, parental education is widely regarded as the most powerful indicator of socioeconomic status (SES) on children’s educational development^8–10^.

One important educational outcome that starts to form during elementary school is academic self-concept (ASC)^11–14^. ASC is a motivational construct that refers to students’ beliefs about their abilities in academic domains^13,15^. Students who have a more positive ASC generally report less anxiety and depression symptoms, are less frequently victimized and excluded by peers, show more classroom engagement as well as higher self-esteem, intrinsic motivation, academic achievement, educational attainment and occupational aspirations^11,16–23^. However, children follow different trajectories in their ASC development^12^, which may put those with lower ASC at risk for less optimal outcomes both during and after their school career. Ideally, all children should have the opportunity to profit from the benefits associated with developing a positive ASC during elementary school. Therefore, longitudinal research is needed to identify the determinants of inequalities in ASC development^12,24^.

Although a part of individual differences in ASC development is attributable to individual child differences in academic achievement (AA) outcomes, such as GPA, grades and test scores^25,26^, parental- and school-related factors seem to contribute to ASC as well^24,27–30^. Determining these factors may be helpful in early identification of children at risk for developing lower ASC. Among such factors, parental education at both the household and school levels and the synergy between these levels might be particularly important^29,31,32^. The current study therefore examined whether household- and school-level parental education independently associated with child- and school-level ASC development from fourth to sixth grade of elementary school (RQ1a). It also examined whether school-level parental education moderated the association between household-level parental education and child-level ASC development (RQ1b). In addition, because AA is a robust predictor of ASC^11,25,33,34^ and parental education is a robust predictor of AA across many studies^8–10,20,35^, we also investigated whether child- and school-level AA (partially) explained the respective associations between household- and school-level parental education and child- and school-level ASC development (RQ2).

The household and school contexts are the most influential environments in elementary school children’s development^36^. In the present study, household-level parental education refers to the education level of children’s parents, while school-level parental education refers to the *per-school percentage* of children with lower-educated parents. According to social-cognitive theory, social environments are key in the development of motivation and learning^37^. Thus, it is possible that parental education inequalities in the household and school environments may lead to differences in children’s ASC development. The “reciprocal interactions” framework of social-cognitive theory explains the dynamic interplay (i.e., bidirectional influences) between personal (e.g., self-beliefs, including ASC), behavioral, and environmental processes (e.g., home, school) in motivation and learning^15,24,37,38^. That is, ASC may be influenced by household- and/or school-level parental education over time.

At the household level, compared to lower-educated parents, higher-educated parents have more access to cultural, social, and economic capital^39–41^ and may thus have more resources and opportunities to foster the educational development of their children. Due to these disparities in (access to) capital, lower-educated parents and higher-educated parents may offer different opportunities for their children. Lareau^41^ proposes that in their approaches to parenting, higher-educated parents use their advantage of capital and engage more in “concerted cultivation,” whereas lower-educated parents engage more in the “accomplishment of natural growth.” The “concerted cultivation” approach prioritizes children’s structured (extracurricular) activities, language and reasoning skill development at home, involvement in children’s schooling, encouragement of questioning and challenging authority, all of which might result in an emerging “sense of entitlement” in children^41^. The “accomplishment of natural growth” approach prioritizes development through everyday experiences, unstructured activities, and free play. In this approach, parents are less active in children’s schooling; rather, they rely more on educators and expect them to be primarily responsible for children’s learning^41^. The differences in capital and resources and subsequently divergent parenting approaches between higher- and lower-educated parents may create differences in the household environments that children grow up in, which may result in children of higher-educated parents developing more positive ASC compared to children of lower-educated parents across the late elementary school years.

At the school level, higher parental education schools (i.e., schools with lower percentages of children of lower-educated parents) may have more material resources, more competitive and advanced curriculum, teachers with higher expectations and ability estimates of students, students with less social-emotional difficulties as well as higher learning motivation and achievement compared to lower parental education schools (i.e., schools with higher percentages of children of lower-educated parents)^3,6,24,42–45^. These differences in the social environments of higher and lower parental education schools may partly explain differences in ASC development. The explanation may go in one of two directions. First, it is possible that ASC levels may develop more positively in higher parental education schools. This may be because of relatively homogenous parental education compositions, such as the more positive motivation and positive teacher contagion or spillover effects in higher parental education than in lower parental education schools. It may also be due to the characteristics associated with lower and higher parental education schools. For example, children’s ASC may be positively influenced by teachers with more motivation or schools with more learning materials (environment). Second, and conversely, it could also be the case that ASC develops more positively in lower parental education schools than in higher parental education schools. This is because previous studies suggest that students in less competitive and lower achieving schools report higher child-level ASC compared to students in more competitive and higher achieving schools^31,46–51^. In line with this, it is possible that the less competitive nature and the less rigorous curriculum of lower parental education schools may also associate with a more positive school-level ASC development.

It is also possible that the ASC development of children of higher- and lower-educated parents may differ depending on whether they attend higher or lower parental education schools. According to the stage-environment fit theory, students feel more motivated in school environments that offer opportunities that fulfill their developmental needs and address their interests and skill level^52^. That is, a poorer stage-environment fit may lead to a less positive development. To give just one example, a previous study examining depression symptoms showed that children growing up in lower-educated households showed a faster growth rate of depression symptoms throughout elementary school when they attended higher but not lower parental education schools^3^. Furthermore, studies on the big-fish-little-pond effect (BFLPE), which is based on social comparison theories, suggest that students in higher achieving schools report lower ASC levels compared to equally talented students in lower achieving schools^46–51,53,54^. In line with these theories and studies, the potential mismatch between the characteristics and expectations of both higher and lower parental education households and schools may lead to different ASC growth patterns. Thus, it is possible that the association between household-level parental education and child-level ASC development may differ in magnitude in higher versus lower parental education schools (i.e., cross-level interaction of household- and school-level parental education).

To date, previous studies examining ASC mostly used cross-sectional data or used adolescent samples^27,29,31,55^. Additionally, to our knowledge, the majority of the studies only looked at child-level ASC as an outcome variable rather than both child- and school-level ASC (but see ref. ^29^). This suggests that previous studies did not jointly consider the household and school levels of social context with respect to parental education and ASC in nested longitudinal study designs across consecutive years in elementary school. Not considering both levels may generate incomplete conclusions because effects may be solely attributed to one level. That is, effects found in previous studies attributed to household level could have been (partially) affected by school level, and vice versa. Previous cross-sectional research—at the household level—showed that youth who had parents with lower education levels, or parents with lower income or occupation status (i.e., other indices of SES) reported less positive ASC^24,27,50,55–58^. In addition, lower SES children were shown to view themselves as less worthy, less deserving, and less capable of growing their intelligence than higher SES children^24^. Although previous studies examined the association between school-level SES and child-level ASC^31,50,59^, to our knowledge—at the school level—only one cross-sectional study examined the associations between school-level SES and school-level ASC. This study used PISA data to test school-level associations among adolescents across different countries and education systems and found mixed results^29^. Similarly, and to the best of our knowledge, only one cross-sectional study examined the interaction between household SES (high, medium, low) and school SES (dichotomous variable) on ASC but found no interaction effects among 6th graders^31^. A study on global self-esteem, a concept related to ASC^30^, found that the positive association between parental education and self-esteem was stronger in higher parental education schools than in lower parental education schools^32^. It therefore remains unknown whether and how the development of child- and school-level ASC differs in higher- and lower-educated households and schools across the late elementary school years (main effects). In addition, it is also unclear whether and to what extent household- and school-level parental education interact to explain differences in child-level ASC development (cross-level interaction effect).

While the first step is to gain an understanding of ASC development of children in higher- and lower-educated households and schools, delving deeper into understanding ASC development requires considering underlying factors that may (partly) explain these associations. To this end, academic achievement (AA) at the individual-child and school levels could serve as mediators. Extant research shows associations between household- and school-level parental education and child- and school-level AA, suggesting higher achievement levels for children growing up in higher-educated households and schools^6–9,35^. Similarly, the role of child-level AA in child-level ASC formation has been well documented in many studies across different countries^25,34,47,60^. These studies show that children who perform better academically are more likely to show more positive ASC. Furthermore, studies show less positive child-level ASC in higher achieving schools than in lower achieving schools (i.e., association between higher school-level AA and lower child-level ASC)^31,48,50,54^. At the same time, when examining the associations between school-level AA and school-level ASC across different education systems among adolescents, the results are not consistent across countries^29^. Therefore, more research is needed to increase our understanding of the associations between parental education, ASC and AA at both the household and school levels of context during the elementary school years. Due to the robust associations reported between household- and school-level parental education and AA^6,7,9,35^, as well as associations between AA and ASC^25,29,34,47^, it is possible for child- and school-level AA to mediate the respective associations between household- and school-level parental education and child- and school-level ASC development. To our knowledge, this has not been tested at both the household and school levels in a multilevel model in elementary school. Yet, attaining a deeper insight into the ASC development of children in higher- and lower-educated households and schools is necessary to identify avenues to decrease inequalities in children’s development. This is especially important in the elementary school period because child-level studies found that the effect of AA on ASC is stronger during childhood than in later life course stages, such as in the adolescence period^25^.

Given the importance of ASC not only as a motivational construct but also for well-being^17,18,25,26^, it is necessary to study whether the differences in ASC development may be partly explained by a robust characteristic of children’s most immediate environments: parental education levels. With the insights gained from this study, it could be determined whether and for which context(s) there is a need for (preventative) interventions to reduce inequalities in ASC development. Therefore, the first research question (RQ1a) examined the association between household-level parental education with the initial level (i.e., intercept parameter) and the development (i.e., slope parameter) of child-level ASC from fourth to sixth grade. Similarly, it examined the association between school-level parental education and the initial level and the development of school-level ASC from fourth to sixth grade. At the household level, we hypothesized that children of lower-educated parents would develop less positive ASC than children of higher-educated parents (negative associations between lower parental education and child-level ASC). The gender of the child was used as a control variable. At the school level, we had competing hypotheses due to the limited and mixed findings in the existing literature^29,31^. Thus, we were agnostic on the direction of the associations. In addition, we also examined whether school-level parental education moderated the association between household-level parental education and the initial level and the development of child-level ASC from fourth to sixth grade (cross-level interaction) (RQ1b). Based on theories and previous research, we tentatively expected the association between household-level parental education and child-level ASC development to be stronger in higher versus lower parental education schools. The second research question (RQ2) tested the mediating role of child-level AA in the association between household-level parental education and the initial level and the development of child-level ASC. Similarly, it also tested the mediating role of school-level AA in the association between school-level parental education and the initial level and the development of school-level ASC. We expected both child- and school-level AA (i.e., standardized (“CITO”) *final* test scores in grade six in the Netherlands) to be significant mediators. To answer our research questions, we used multilevel latent growth models (ML-LGM) with a two-level time-nested-within-individual data structure (level 1 = variation across individual children/household, level 2 = variation across schools) in a sample of 679 (51% girls) children from 18 Dutch elementary schools who were followed annually from fourth to sixth grade. In addition, to ensure that the results were robust despite missing data, we conducted sensitivity analyses.

## Results

### Descriptive statistics of the study variables and the development of ASC

Descriptive statistics of household- and school-level parental education are in Table 1. The correlation between household- and school-level parental education was positive (*r* = 0.32, *p* < 0.001), indicating a tendency toward relatively similar parental education attainment at both levels.

**Table 1:** Descriptive statistics of household- and school-level parental education

Household-level parental education *(N* = 679)	*N* (%)	School-level parental education (*N* = 18)	%
No education/early education	1 (0.1%)	Range	0.1–44.9%
Primary education	3 (0.4%)	Mean	6.3%
Lower secondary education	20 (2.9%)	Standard Deviation	10.4%
Upper secondary education	26 (3.8%)	Median	1.9%
Post-secondary non-tertiary education	19 (2.8%)		
Short-cycle tertiary education	135 (19.9%)		
Bachelor’s or equivalent degree	217 (32.0%)		
Master’s degree, equivalent or higher	258 (38.0%)		

The range of household-level parental education scale is from 0 (no education/early education) to 7 (master’s degree, equivalent or higher). School-level parental education could range from 0 to 100%, with higher percentage scores indicating a higher concentration of children of lower-educated parents (see “Methods” section for details).

Results from unconditional models (models without predictors or covariates) showed that, on average, ASC levels, which could range from 0 to 3, were relatively high across the three studied years (*M*_intercept_ = 2.19, *p* < 0.001; σ^2^_intercept, within_ = 0.175, *p* < 0.001; σ^2^_intercept, between_ = 0.003, *p* = 0.005). Results showed stable ASC levels from fourth to sixth grade, as shown by the non-significant slope parameter mean (*B* = 0.00, *p* = 0.76). Furthermore, compared to girls, boys reported moderately higher levels of ASC in fourth and fifth grades (*t*(661) = −3.36, *p* < 0.001, *d* = 0.53; *t(*660) = −2.41, *p* = 0.016, *d* = 0.53, respectively) but not in sixth grade, *t(*649) = −1.26, *p* = 0.208, *d* = 0.54. Child-level AA in sixth grade ranged from 511 to 550 (*N* = 339, *M*_*CITO final test-score*_ = 537, SD = 8, median = 537). School-level AA in sixth grade ranged from 531–537 (*N* = 14, *M*_*CITO final test- score*_ = 535, SD = 1.92, median = 535). There were no significant differences between boys and girls in their AA levels (*t*(339) = −0.100, *p* = 0.623).

### Research Question 1a (RQ1a): Main effect associations of household- and school-level parental education with child- and school-level ASC

#### Model building

The final conditional models were built only using ASC intercept parameters. That is, in the final models, we removed the ASC latent slope parameters and only included ASC latent intercept parameters. This was done for the following reasons: First, the rate of change of ASC levels from fourth to sixth grade was virtually zero and not significant (*B* = 0.00, *p* = 0.76). Second, the associations between our predictors and slope parameters of child- and school-level ASC were never significant. Last, the inclusion of the ASC latent slope parameters resulted in a saddle-point parameter vector. Thus, ASC latent slope parameters were removed from all final models. Nevertheless, it should be noted that when ASC latent slope parameters were included in the models, the parameter results of all models pointed toward the same results. These initial models that included the slope parameters are available in OSF (see the link under “Code availability”). Due to the reasons outlined above, we proceed with only presenting the effects on the ASC latent intercept parameters. Model fit indices of the model with only ASC latent intercepts parameters were acceptable (RMSEA = 0.079, CFI = 0.952, TLI = 0.974, SRMR_within_ = 0.042, SRMR_between_ = 0.013).

#### Results RQ1a

**Fig. 1:**
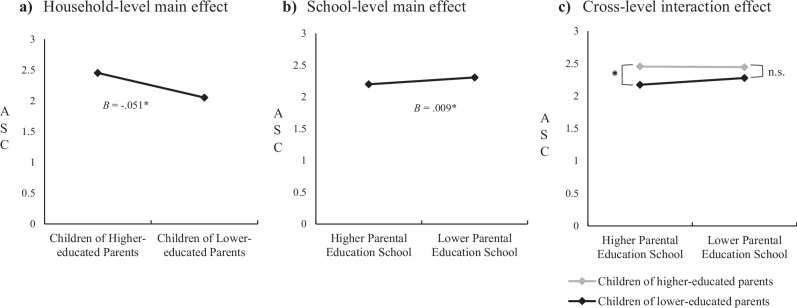
Main effect associations of and cross-level interaction between household- and school-level parental education on ASC. **a** Household-level main effect. **b** School-level main effect. **c** Cross-level interaction effect. *p ≤ 0.01. **a** Main effect association of household-level parental education on child-level ASC. **b** Main effect association of school-level parental education on school-level ASC. **c** Cross-level interaction between household- and school-level parental education on child-level ASC (the gray line represents ASC levels of children of higher-educated parents and the black line represents the ASC levels of children of lower-educated parents). The scale of ASC ranges from 0 to 3, with higher scores indicating higher ASC levels. For illustrative purposes only, in the figures, children of higher-educated parents represent children whose parents completed bachelor’s degree or equivalent level and children of lower-educated parents represent children whose parents completed lower secondary education or equivalent level. Furthermore, lower and higher parental education school percentages were calculated by 0.5 SD above and below the mean of school-level parental education percentage scores.

The results of the main effect associations can be seen in Table 2 and Fig. 1a, b. The main effect results showed that household-level parental education was significantly associated with the ASC latent intercept parameter in fourth grade. That is, children of lower-educated parents reported less positive views about their academic abilities than children of higher-educated parents in fourth grade, and these differences remained stable from fourth to sixth grade. Furthermore, school-level parental education was significantly associated with the school-level ASC intercept parameter, suggesting that school-level ASC scores were lower in higher parental education schools than in lower parental education schools. In other words, children in lower parental education schools, on average, viewed their academic abilities more positively than children in higher parental education schools from fourth to sixth grade.

**Table 2:** Main effect associations of and cross-level interaction between household- and school-level parental education on ASC

	Academic self-concept intercept (Grades 4–6)				
	β	*B*	S.E.	*p*-value	CI (95%)
**Main effect model**					
**Within** **(Child/household level)**	Child-level academic self-concept				
Gender	0.288	0.120	0.048	0.012	0.027, 0.213
Household-level parental education	−0.138	−0.051	0.020	0.010	−0.089, −0.012
**Between** **(School level)**	School-level academic self-concept				
School-level parental education	0.863	0.009	0.002	<0.001	0.004, 0.014
**Cross-level interaction model**					
	Child-level academic self-concept				
Intercept random intercept	-	−0.042	0.022	0.052	−0.085, 0.000
Household- x School-level parental education	-	0.003	0.001	0.015	0.001, 0.005

*N* = 679. Household-level parental education scores were reverse-coded so that higher scores indicate lower household-level parental education. Random intercept: the association between household-level parental education and child-level ASC. School-level parental education scores could range from 0 to 100%, with higher percentage scores indicating lower school-level parental education. Note that the effect of school-level parental education represents the effect at 1% change in school-level parental education. Standardized regression coefficients are not available in MPLUS when testing cross-level interactions with the MLR estimator.

### Research Question 1b (RQ1b): School-level parental education as a moderator of the association between household-level parental education and child-level ASC (cross-level interaction)

Cross-level interaction was also only performed on the intercept parameter of child-level ASC (see “Methods” for reasoning). Results showed significant cross-level interactions between household- and school-level parental education on the child-level ASC intercept parameter (see Table 2). To illustrate the interaction effect (see Fig. 1c), we probed the interaction effects using 0.5 SD above (schools with ~11% of children with lower-educated parents) and below (schools with ~1% of children with lower-educated parents) the mean of school-level parental education. Results showed that children of lower-educated parents reported less positive ASC levels than children of higher-educated parents in higher parental education schools (*B* = −0.056, S.E. = 0.025, *p* = 0.026, CI [−0.106, −0.007]), while in lower parental education schools, there was no significant association between household-level parental education and child-level ASC (*B* = −0.027, S.E. = 0.020, *p* = 0.174, CI [−0.067, 0.012]). In sum, results cautiously suggested that, in terms of their ASC, children of lower-educated parents benefited slightly more from attending lower parental education schools in the last three years of elementary school.

Two sensitivity tests were conducted on multilevel models with 25 imputed datasets and on single-level models (please see “Methods” for further explanation). The sensitivity test results of RQ1a and RQ1b are presented in the Supplementary Note 2 and Supplementary Tables 1, 3, and 5. The results from the single-level models (models without school level) generally lead to qualitatively similar conclusions as the results presented above. The results from the models with imputed datasets showed similar conclusions at the household level. However, the association between school-level parental education and school-level ASC and the cross-level interaction were no longer significant in multilevel models with imputed datasets.

### Research Question 2 (RQ2): Child- and school-level AA as mediators of the associations between household- and school-level parental education and child- and school-level ASC

**Fig. 2:**
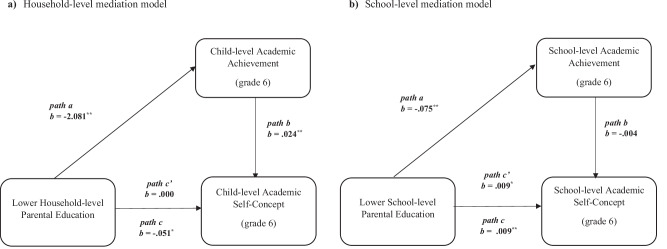
Mediation models at the household and school levels. **a** Household-level mediation model. **b** School-level mediation model. *p < 0.05. **p < 0.001. **a** Household-level mediation model: child-level AA as a mediator of the association between lower household-level parental education and child-level ASC intercept parameter. **b** School-level mediation model: school-level AA as a mediator of the association between lower school-level parental education and school-level ASC intercept parameter. *path c* = total effect (the association between the predictor and the outcome variable in the model without the mediator). *path a* = the association between the predictor and the mediator variable. *path b* = the association between the mediator and the outcome variable. *path c*’ = direct effect (the association between predictor and the outcome variable due to the mediator).

We tested whether child- and school-level AA measured in sixth grade mediated the associations between household- and school-level parental education and child- and school-level ASC intercept parameters in sixth grade. Mediation analyses were tested at both household and school levels in the same model. At the household level, household-level parental education was the predictor variable, child-level AA was the mediator, and child-level ASC intercept parameter was the outcome variable. At the school level, school-level parental education was the predictor variable, school-level AA was the mediator and school-level ASC intercept parameter was the outcome variable. Results of the model with child-level and school-level AA as mediators can be seen in Table 3 and in Fig. 2a, b.

**Table 3:** Mediation model and mediation and interaction model: estimates of the associations between household- and school-level parental education, child- and school-level academic achievement and child- and school-level ASC in sixth grade

	Academic achievement (Grade 6)					Academic self-concept (Grade 6)				
	β	*B*	S.E.	*p*-value	CI (95%)	β	*B*	S.E.	*p*-value	CI (95%)
**Mediation model**										
**Within** **(Child/household level)**	Child-level academic achievement					Child-level academic self-concept				
Gender	0.084	0.671	0.529	0.205	−0.367, 1.708	0.250	0.104	0.049	0.035	0.007, 0.201
Household-level parental education	−0.295	−2.081	0.287	<0.001	−2.643, −1.519	0.001	0.000	0.021	0.986	−0.041, 0.041
Child-level academic achievement	-	-	-	-	-	0.471	0.024	0.003	<0.001	0.019, 0.030
**Between** **(School level)**	School-level academic achievement					School-level academic self-concept				
School-level parental education	−0.421	−0.075	0.021	<0.001	−0.117, −0.033	0.833	0.009	0.004	0.014	0.002, 0.016
School-level academic achievement	-	-	-	-	-	−0.072	−0.004	0.025	0.864	−0.054, 0.045
						Child-level academic self-concept				
Indirect effect (mediation)	-	-	-	-	-	-	−0.051	0.010	<0.001	−0.070, −0.032
**Mediation and cross-level interaction model**										
Household- x School-level parental education	-	-	-	-	-	-	0.003	0.001	0.028	0.000, 0.005
Indirect effect (mediation)	-	-	-	-	-	-	−0.048	0.009	<0.001	−0.066, −0.029

*N* = 679. Household-level parental education scores were reverse-coded so that higher scores indicate lower household-level parental education. School-level parental education scores could range from 0 to 100%, with higher percentage scores indicating lower school-level parental education. Note that the effect of school-level parental education represents the effect at 1% change in school-level parental education. Standardized regression coefficients are not available in MPLUS when testing cross-level interactions with the MLR estimator.

At the household level, results showed that child-level AA mediated the association between household-level parental education and child-level ASC in sixth grade (see Fig. 2a). That is, a significant indirect effect of household-level parental education on child-level ASC intercept parameter was found through child-level AA (*B* = −0.051, S.E. = 0.010, *p* < 0.001, CI [−0.070, −0.032]). Results indicated that, as compared to children of lower-educated parents, children of higher-educated parents showed higher AA and therefore reported more positive ASC levels in sixth grade.

At the school level, school-level AA was not a mediator of the association between school-level parental education and school-level ASC intercept parameter (see Fig. 2b). School-level parental education was significantly associated with both school-level AA and school-level ASC. That is, compared to higher parental education schools, in lower parental education schools, the average ASC levels were higher, but the average AA scores were lower. However, the association between school-level AA and school-level ASC was not significant.

The sensitivity analyses for RQ2 were performed on imputed datasets, single-level models, and on a subsample with complete academic achievement data. Sensitivity test results of RQ2 are presented in the Supplementary Note 2 and in Supplementary Tables 2, 4, 6 and 7. The mediation results lead to qualitatively similar conclusions as the results presented above. Furthermore, the results of the cross-level interactions examined within the model of RQ2 are described in the Supplementary Note 3.

## Discussion

The elementary school period is critical to promote equal opportunities for all children. The effects of parental education inequalities at both the household and school levels on children’s development have been documented in a wide range of outcomes, including outcomes in educational settings^3,6,7,9^. However, most of these studies have been cross-sectional in nature. To better understand the associations of parental education with children’s educational and motivational development and to potentially guide interventions in educational settings, this study focused on academic self-concept (ASC) development in elementary school. Specifically, this study, to the best of our knowledge, was the first to longitudinally examine the main effect and cross-level interactions of household- and school-level parental education on ASC development from fourth to sixth grade of elementary school. In addition, in order to better understand the (potential) associations between household- and school-level parental education and child- and school-level ASC, we tested whether child- and school-level academic achievement (AA) mediated the associations between household- and school-level parental education with child- and school-level ASC development.

Overall, the children in our sample reported relatively high and stable ASC levels from fourth to sixth grade. This is an important finding in itself because positive ASC has benefits for educational and mental health outcomes^11,13,16–18,20,26^. Our results showing the stability of ASC levels in the last three years of elementary school are in line with some previous research but not with others that showed an increase or a decrease in ASC levels^12,61–63^. These differences in ASC development rates may be due to the country-level differences in educational systems^29^. It could also be that motivational variables such as ASC may be dynamic after school transitions, such as from elementary to secondary school, and that the last years of elementary school may be a period of stability.

At the household level, results from RQ1a showed significant associations between household-level parental education and ASC from fourth to sixth grade. Children of lower-educated parents viewed their academic abilities less positively than children of higher-educated parents. Furthermore, contrary to our hypothesis, the development of ASC was stable from four to sixth grade and similar for children of higher- and lower-educated parents. Results from RQ2 provided deeper insights into the varying ASC levels between children of higher- and lower-educated parents. That is, as compared to children of higher-educated parents, children of lower-educated parents had lower academic achievement scores and in turn reported less positive ASC levels in sixth grade. These findings suggest that child-level AA is one of the underlying mechanisms that could partly explain the differences in ASC of children of higher- and lower-educated parents. The findings support previous research, which showed similar associations between household-level parental education and child-level AA^8,9^ as well as child-level AA and child-level ASC^11,13,25^. Our findings extend previous research by (1) showing how household-level parental education longitudinally contributed to ASC from fourth to sixth grade and by (2) identifying the explanatory role of AA in ASC of children growing up with higher- and lower-educated parents.

The findings at the household level might be related to parental practices and the education system in the Netherlands. The Netherlands has a tracked educational system in which children transfer to various and specific secondary education track levels at the end of elementary school (the sixth grade). Track decisions for each child are based on standardized test results (i.e., AA (“CITO”) *final* test scores used in this study) and teacher recommendations, which start to become relevant for children’s future educational track in the vast majority of Dutch elementary schools. This makes the last three years of elementary school especially important for children’s future opportunities in secondary and even in tertiary education. One might speculate that differences in parenting approaches (e.g., “concerted cultivation” and “accomplishment of natural growth”^41^) may be related to the differences found in these years. Higher-educated parents may use the advantages of their capital not only to influence numerous aspects of their children’s schooling but also to influence their children’s achievement in the hopes that their children transfer to highly tracked secondary education levels. Therefore, higher-educated parents’ parenting approach and capital advantage coupled with their higher educational aspirations for their children^8,9^ could explain how parental education contributes to their children’s achievement levels. This in turn could explain children of higher-educated parents’ more positive beliefs about their academic abilities. In line with previous studies^11,13,25^, children who achieve higher AA scores are likely to view their academic abilities more positively than those who achieve lower AA scores. This could especially be the case towards the end of the elementary school period: a time period in which achievement scores become increasingly important in tracked education systems like that of the Netherlands.

At the school level, the results from both RQ1a and RQ2 showed that ASC levels were lower in higher parental education schools than in lower parental education schools. In addition, the development of ASC was similar across schools. These results are notable insofar as previous literature often suggested less positive developmental outcomes for children attending lower parental education schools^3,6,35,64^. The frame of reference model suggests that children compare themselves to others in their immediate surroundings^65^. This may especially be the case for the young (i.e., elementary school) children in our study whose frame of reference for ASC is largely confined to experiences within their schools^31^. To our knowledge, there was only one study that investigated school-level SES and school-level ASC associations^29^. That study used PISA data of adolescents and examined the school-level associations in different countries and education systems. Their results showed non-significant associations in some countries and significant associations between higher school SES and higher school-level ASC in other countries^29^. Our findings may provide novel insights into the school-level SES and school-level ASC associations in the last three years of elementary school in the Dutch context.

Furthermore, results from RQ2 showed that school-level AA was not an explanatory factor (i.e., mediator) in the association between school-level parental education and school-level ASC. It is possible that the often-reported characteristics, other than higher average achievement, of higher SES or parental education schools, such as the more competitive environment and rigorous curriculum, may have contributed to the lower ASC levels in these schools^46^. It could also be that in higher parental education schools, children may be more critical or skeptical of their academic abilities. However, when addressing missing data via multiple imputations, the previously significant association between school-level parental education and school-level ASC disappeared. This threatens the robustness of our results and calls for a careful interpretation of these specific findings. Thus, future replication research is warranted prior to reaching firm conclusions.

Our school-level mediation hypothesis was not supported. While we found robust associations between school-level parental education and school-level AA, the association between school-level AA and school-level ASC was not significant. The latter finding is not in the same direction as previous studies that reported lower child-level ASC in higher achieving schools^46,47,49,50,53^; however, our findings complement these previous studies, which examined child-level ASC, by examining school-level ASC as an outcome. Our results partly support the abovementioned PISA study that also examined the associations between school-level AA and school-level ASC across different countries and education systems^29^. Associations were reported to differ between countries, ranging from non-significant, significant and positive, to significant and negative^29^. While previous research shows robust associations between higher school-level AA and lower child-level ASC^47,49–51^, the association between school-level AA and school-level ASC may not be as robust and may be context dependent (i.e., effects may depend on the country and education system).

Results from cross-level interactions (RQ1b) showed that school-level parental education moderated the association between household-level parental education and child-level ASC. These results suggested that children of lower-educated parents, in terms of their ASC, may benefit more from attending lower parental education schools than attending higher parental education schools. That is, children of lower-educated parents seem to view their academic abilities more positively in lower than in higher parental education schools. In this way, results for children of lower-educated parents may cautiously support stage-environment fit theory and social comparison theories, such as the frog pond perspective and are in line with findings of previous studies showing more difficulties for children of lower-educated parents who attend higher parental education schools^3,66^. These results contradict the null findings of a previous single-level study among sixth graders^31^. However, the (small) interaction effects should be interpreted with caution, especially since the results from the models with multiple imputations did not yield robust findings regarding the significant interaction effects.

The limitations of this study should be considered while interpreting the results. First, we had a convenience sample. The average education levels of the parents in this study were higher than that of the general Dutch population^67^. Selective attrition could have been in play at both the household and school levels. Second, we had a small number of clusters/schools in our multilevel models. Third, it is noteworthy that our preregistered inclusion criteria required participants to have household- and school-level parental education data and two years of ASC data. Participants who did not meet these criteria were excluded from the main analyses. We had a large proportion of missing data, particularly for parental education (see “Methods”). Overall, included and excluded participants *and* participants with and without missing data did not always differ on all constructs, and if they did, the differences were generally small (see Supplementary Note 1). In the main manuscript, we adhered to the methodological approach described in our preregistration due to the large amount of missing data of our main predictors that are vital to our research questions. Nevertheless, we conducted sensitivity tests to address the missing data. The household-level associations and mediation results at both household and school levels were robust. Please note that we also had missing data on our mediator, which was academic achievement. The child-level AA data were obtained from schools, and not all schools gave consent or provided this data. This may suggest selectivity by schools, especially considering that the schools children with and without AA data attended differed in their parental education and achievement compositions. Therefore, this subsample may not be representative of the larger final sample due to the differences between children with and without AA data. Furthermore, the results of the school-level main effects and cross-level interactions did not always yield similar conclusions. Thus, these specific results should be interpreted with caution. Fourth, we only had child- and school-level AA data in sixth grade. Yet, our AA measure was based on standardized *final* test scores and thus was a more comparable measure than GPAs or grades of children from different schools. Therefore, firm conclusions should not be made before replication studies with larger cluster numbers, longitudinal child- and school-level AA data, and more variability in parental education at both levels are conducted. Fifth, it is noteworthy that a bidirectional relationship exists between AA and ASC^25,34^. Although recent studies showed that AA is a stronger predictor of ASC than vice versa^25,34^, it has been shown that ASC could also be a mediator of the association between SES and AA (and other self-belief measures) at the household level^68^. School-level ASC as a mediator could also be examined in future studies. Furthermore, future research is encouraged to juxtapose the contextual effects^50^ to uncover the unique effects of parental education and academic achievement on ASC development at both the household/child and school levels in elementary school. Last, due to data unavailability, we were not able to include additional SES indicators or control for factors often correlated with parental education at both the household (e.g., income, household wealth, number of books, extracurricular activities) and school (e.g., teacher motivation, school climate, peer competitiveness) levels. Thus, our results do not imply any causal associations nor can they lead to firm conclusions, but they seek to invite further investigations.

The insights gained from this study may indicate a need for interventions to nurture ASC of children of lower-educated parents and children in higher parental education schools. While more research is needed at the school level, our results suggest that one way to nurture the ASC development of children of lower-educated parents is to provide them with academic achievement support during elementary school. In general, results also point toward a need for stronger academic support in lower parental education schools. To conclude, effective ASC interventions while children are still in elementary school may help to maintain the high levels of ASC observed and to potentially reduce the relative differences in ASC levels between children growing up in higher- and lower-educated contexts. This may additionally be important because ASC levels not only decrease from elementary to secondary school but also show a gentler decline for higher SES compared to lower SES children^69^. With such efforts, children may learn the skills to nurture a positive ASC not only during elementary school but also when they enter new environments (e.g., secondary education) and thereby enjoy the benefits of positive ASC.

## Methods

Please note that RQ1 was preregistered in OSF (https://osf.io/uabe8/?view_only=a21f42ad071945afb6888b5eaa98bc1e); however, RQ2 was added after peer review.

### Participants

Data came from the larger longitudinal project “Happy Children, Happy Adolescents? (HCHA)” that aimed to investigate emotional, behavioral, social, and cognitive development of children. Children were recruited from elementary schools located in the Netherlands. The *preregistered inclusion criteria* of this study included parental consent, children to be enrolled in one of the participating schools, data on household- and school-level parental education, and self-reports of at least two years of repeated measures of ASC from fourth to sixth grade.

Of the 1617 participants who consented to participate in the larger HCHA project, 883 (54.6%) participants did not have parental education data. This means that of all participants, we had 734 (45.4%) participants with available parental education data. Furthermore, 151 (9.3%) had two waves, and 218 (13.5%) had one wave of missing ASC data. Of all participants, 601 (37.2%) had available child-level AA data. Out of 22 schools, school-level AA scores were available for 16 schools (72.2%) and school-level parental education data were available for 21 schools (95.5%).

Of the 734 participants with available parental education data, 24 (3.3%) had missing school-level parental education data, and of the remaining 710 participants, 31 (4.4%) had two missing waves of ASC data. This meant that 55 participants with parental education data were excluded from the final sample. Therefore, following the preregistered inclusion criteria, the final sample resulted in 679 participants (51% female, 84% ethnic Dutch background) from 18 schools. From the 679 participants who were included in the final sample, 339 (49.9%) had available child-level AA data, and 14 (77.8%) schools had available school-level AA data. The comparisons between (a) children with and without missing data who consented to participate in the larger HCHA study on all constructs and (b) excluded versus included children in the final sample based on the preregistered inclusion criteria are carefully presented in detail in the Supplementary Note 1. The differences between included and excluded participants *and* participants with and without missing data were not always found on all constructs, but when they were found, these were generally small (except for some differences regarding AA).

### Procedure

The first schools that agreed to participate were included in the study. Informed consent for participation was obtained from parents in accordance with the Declaration of Helsinki. Parents were informed about the project each year and gave active consent at study entry and passive consent in the following years for their children’s participation. Parents and children could revoke their consent at any time. Data from children were obtained annually from the spring of fourth grade until the spring of sixth grade between 2015 and 2019. During the data collection day, children responded to questionnaires using tablets in their classrooms and were supervised by trained research assistants. Data on household-level parental education were obtained from parents via online questionnaires. Data on child-level AA were obtained from the participating schools. Data on school-level parental education and school-level average AA are publicly available (www.duo.nl). Ethical approval for study procedures was obtained from the Medical Ethics Committee of the Vrije Universiteit Amsterdam Medical Center, The Netherlands [protocol number: NL37788.029.1].

### Measures

Household-level parental education: Parents reported their highest level of completed education. These levels were rated according to the Dutch Standard Education Classifications^70^, which is in line with the International Standard Classification of Education (ISCED)^71^. Following the ISCED classifications, the parental education levels were coded using an 8-point scale, with education levels ranging from 0 = no education/early education, 1 = primary education, 2 = lower secondary education, 3 = upper secondary education, 4 = post-secondary non-tertiary education, 5 = short-cycle tertiary education, 6 = bachelor’s degree or equivalent, to 7 = master’s degree, equivalent or higher. Following other studies^3,8,64,72^, parental education scores were based on the highest completed parental education level per household. The parental education levels were reverse-coded so that higher scores indicated lower parental education levels for ease of interpretation. Note that the correlation between the level of education of the father and the mother was positive (*r* = 0.55, *p* < 0.001), indicating a tendency toward similar maternal and paternal attained education.

School-level parental education: School-level parental education was based on the per-school percentage of children of low-educated parents. Thus, in this study, school-level parental education was based on the percentage score of low parental education levels of children within each participating school and not just the children participating in the present study. Low-education refers to either both parents completing no more than elementary school education or one parent completing no more than elementary education and the other parent completing no more than lower-level secondary education (i.e., practical training or basic/middle-management pathway of preparatory vocational secondary education)^73^. The percentage scores could range from 0 to 100%, with higher percentage scores indicating schools with higher percentages of children of low-educated parents. This information is publicly available (www.duo.nl).

Academic self-concept: The Dutch adaptation (Competentie Belevingsschaal voor Kinderen; CBSK^74^) of the academic self-concept subscale of the Self-Perception Profile for Children (SPPC^75^), which is the revised version of the Perceived Competence Scale for Children (HPCS^76^), was used. The Dutch ASC subscale has 6 items (i.e., “I am smart,” “I am very good at schoolwork,” “I finish schoolwork quickly,” “I can easily remember everything I learn in school,” “I do very well at school,” “At school, I almost always know the answers of the questions”) and uses a four-point Likert scale ranging from 0 (not true), 1 (a little not true), 2 (a little true) to 3 (true). Cronbach’s alpha ranged from 0.82 to 0.84 across the three studied years. Higher scores indicated higher levels of ASC. The Dutch translation (CBSK) has been shown to have good psychometric properties^77,78^.

Child-level academic achievement: Child-level academic achievement was measured by standardized test scores. Specifically, we used “CITO” *final* test scores, which are used in the Dutch educational system to inform the advised secondary school tracks that children will follow upon the conclusion of elementary school. The CITO *final* test includes questions on mathematics, language, and information processing. It is administered in sixth grade (last year of elementary school), and one *final* score is calculated for each student. The scores could range from 501 to 550, with higher scores indicating higher academic achievement scores (www.cito.nl).

School-level academic achievement: School-level academic achievement was based on the average CITO *final* test scores per school in sixth grade. This means that the average CITO final test scores were based on test scores of all children in our participating schools and were not only based on the scores of the children participating in the present study. This information is publicly available and was obtained from the database of the Ministry of Education, Culture, and Science (www.duo.nl). The scores could range from 501 to 550, with higher scores indicating higher average school-level academic achievement scores (www.cito.nl).

### Analysis strategy

Multi-level latent growth models (ML-LGM) with two-level time-nested-within-individual data structure (1 = variation across individual children/household, 2 = variation across schools) were used to test our hypotheses. The development of child- and school-level ASC was estimated by latent intercept and latent slope parameters. The latent intercepts represented child- and school-level ASC in fourth grade (baseline) and latent slopes represented the rate of change (development) of child- and school-level ASC from fourth to sixth grade. All models were fitted in Mplus.

Before testing our hypotheses, we first examined whether there was a general (increase, decrease, stable) development of ASC using unconditional ML-LGMs (i.e., ML-LGMs without covariates). Then, to answer RQ1a, we tested main effects by examining the independent associations of household- and school-level parental education with ASC latent growth parameters using ML-LGM. That is, at the within level (level 1), child-level ASC intercept and slope parameters were regressed on household-level parental education. At the between level (level 2), school-level ASC intercept and slope parameters were regressed on school-level parental education. To answer RQ1b, which tested whether school-level parental education moderated the association between household-level parental education and child-level ASC development, we conducted cross-level interactions. However, we first checked whether such interactions could be performed. That is, at the within level, we modeled a random intercept and random slope in which child-level ASC intercept and slope parameters were regressed on household-level parental education. Then we estimated variances of these random intercept and random slopes at the between level. Using Satorra-Bentler Chi-Square Difference tests with Loglikelihood, we checked whether adding a random intercept and/or random slope improved the model fit of the main effect model. Fitting the random intercept (χ^2^(1) = 5.64, *p* = 0.02) but not both random intercept and random slope (χ^2^(1) = 0.37, *p* = 0.54), improved the model fit of the main effect model, indicating that only the association between household-level parental education and child-level ASC intercept parameter varied between schools. Thus, only cross-level interaction of the random intercept was performed. When significant, the cross-level interactions were probed to understand the associations between household-level parental education and child-level ASC development in higher (*M* − 0.5 SD) and lower parental education schools (*M* + 0.5 SD). To probe the interaction effects, we used 0.5 SD because 1 SD below the mean of school-level parental education is less than 0% in our study, which is not within the possible ranges of school-level parental education.

To answer our second research question (RQ2), we examined the mediating roles of child- and school-level AA in the associations between household- and school-level parental education and child- and school-level ASC development. To this end, mediation models were fitted at both the household and school levels in the same model. That is, child- and school-level ASC intercept and slope parameters were regressed on household- and school-level parental education, respectively. Furthermore, child- and school-level AA were regressed on household- and school-level parental education, respectively. In addition, child- and school-level ASC parameters were regressed on child- and school-level AA, respectively. The significance of the indirect effects (a*b) was estimated in Mplus. It should be noted that we had child- and school-level AA data in sixth grade. Therefore, child- and school-level ASC intercept parameters in the mediation models refer to the ASC levels in sixth grade, and the slope parameters retrospectively represent the rate of change of ASC from fourth to sixth grade.

MLR estimators (maximum likelihood estimation with robust standard errors) were used to account for the possible non-normal distribution of data. Deviations from normality were all within the normal range of ASC values from fourth to sixth grade (Skewness range = −0.795 to −0.860; Kurtosis range = 0.994–1.488) and AA values in sixth grade (Skewness_child-level_ = −0.584, Skewness_school-level_ = 0.107; Kurtosis_child-level_ = −0.138 Kurtosis_school-level_ = −0.900). The missing academic self-concept and academic achievement data were handled using Full Information Maximum Likelihood (FIML) estimations. Gender (0 = girl, 1 = boy) was added as a control variable at the within level. Household-level parental education and child-level AA were group-mean centered, and school-level parental education and school-level AA were grand-mean centered to ease the interpretations of our findings. Model fit indices Comparative Fit Index (CFI) and Tucker Lewis Index (TLI) with critical values ≥0.90, Root Mean Square Error of Approximation (RMSEA, critical value ≤ 0.08) and Standardized Root Mean Residual (SRMR, critical value ≤ 0.08)^79–81^ were used to determine model fit. Mplus code and output files are available in OSF (see the link under “Code availability”).

Three types of sensitivity tests (not preregistered, but recommended by reviewers) were conducted to check the robustness of our results. The first model utilized multiple imputations to address missing data. We imputed values for the missing data of household- and school-level parental education, ASC, child- and school-level AA. We ran all the models with 25 imputed datasets. The second model was a single-level model (model without the school level). This model did not have a multilevel structure and used child-level academic self-concept (ASC) as the outcome variable (rather than *both* child- and school-level ASC). In addition to the predictors at the child/household level, this model also included school-level variables (school-level parental education and school-level academic achievement) to predict child-level ASC. These tests were run because we had a relatively small number of clusters. We ran these single-level analyses using both the final sample based on the preregistered inclusion criteria (*N* = 679) and the imputed sample (*N* = 1617). The third model was based on a subsample that only included participants with complete AA data (*N* = 339). This model tested RQ2 to investigate whether child- and school-level AA were significant mediators in this subsample. The results of the sensitivity analyses are presented in the Supplementary Note 2 and in Supplementary Tables 1–7. It should also be noted that to further assess the robustness of our findings, in addition to the sensitivity tests described above, we also conducted two additional types of sensitivity analyses: (1) imputing the missing values for all variables except for ASC using 25 imputed datasets, and (2) applying mean imputation to impute the missing data for all variables except for ASC. Although we do not report the results of these two additional sensitivity analyses in the main manuscript or in the Supplementary Material, the Mplus output files of all sensitivity analyses are available in OSF. Overall, the results across all sensitivity tests lead to similar conclusions as reported in this manuscript.

## Supplementary information

Supplementary Material_ASC_R3_FINAL_SUBMIT

## Data Availability

The data are not currently publicly available due to data privacy/ethical restrictions. The dataset used in this study can be made available upon reasonable request to the corresponding author with a formal data-sharing agreement. Data on school-level parental education and school-level academic achievement are publicly available (www.duo.nl).

## Code availability

The Mplus code and output files are available and accessible in OSF (https://osf.io/xwdsv/?view_only=ffce0a4fc3ed493dabc118fe762d8a54).
