# Supplementary material for: Household- and school-level parental education and academic self-concept development in elementary school
Source: NPJ Sci Learn. 2025 Aug 26;10:62. doi: 10.1038/s41539-025-00354-x (PMC12379102; doi:10.1038/s41539-025-00354-x)
Supplement: Supplementary file 1 — Supplementary Material_ASC_R3_FINAL_SUBMIT [file 41539_2025_354_MOESM1_ESM.pdf]

Supplementary Material

Supplementary Note 1

Supplementary Results on the Proportion and Selectivity of (Missing) Data

1. Comparisons between children with and without missing data who consented to participate in the larger HCHA project (without considering the preregistered inclusion criteria for the present study) ($N = 1617$)

Comparisons regarding children with and without missing parental education data and study variables:

From 1617 children who consented to participate in the larger study, 734 children had available parental education data and 883 children did not have parental education data.

Age and gender: The children without parental education data ($M_{years} = 10.08$, $SD_{\text{years}} = .50$) were on average slightly older than the children with parental education data ($M_{years} = 9.98$, $SD_{\text{years}} = .40$) at baseline, $t(1473) = -4.276$, $p < .001$, Cohen's $d = .223$. There were no differences between these two groups of children with respect to gender distribution, χ^2 (1) = 1.24, $p = .266$.

Academic self-concept (ASC): The ASC levels of children without parental education data did not significantly differ from the children with parental education data in fourth, fifth and sixth grades ($t(1455) = .254$, $p = .800$, Cohen's $d = .013$; $t(1449) = .931$, $p = .352$, Cohen's $d = .049$; ($t(1421) = .925$, $p = .355$, Cohen's $d = .049$, respectively).

Child- and school-level academic achievement: Children without parental education data had lower child-level academic achievement scores and were in schools with lower average achievement scores than children with parental education data ($t(559) = 4.962$, $p < .001$, Cohen's $d = .408$; $t(992) = 15.21$, $p < .001$, Cohen's $d = .969$, respectively).

School-level parental education: Children without parental education data were in schools with slightly higher percentage of children with lower-educated parents than children with parental education data ($t(1568) = -3.029$, $p = .002$, Cohen's $d = .154$).

Comparisons regarding children with and without missing academic achievement data and study variables:

From 1617 children who consented to participate in the larger study, academic achievement data was available for 601 and not for 1016 children.

Age and gender: Children without academic achievement data (M_{years} = 10.05, SD_{years} = .49) and children with academic achievement data (M_{years}= 10.01, SD_{years} = .41) were similar in their age at baseline, $t(1473) = 1.330, p = .184$, Cohen's $d = .072$. There were no differences between children with respect to gender distribution, χ^2 (1) = .205, $p = .651$.

Academic self-concept (ASC): Children without academic achievement data showed slightly higher levels of ASC in fourth grade but similar levels in fifth and sixth grades compared to children with academic achievement data ($t(1455) = 2.353, p = .019$, Cohen's $d = .128$; $t(1449) = .605, p = .545$, Cohen's $d = .033$; $t(1421) = 1.527, p = .127$, Cohen's $d = .082$, respectively).

Household- and school-level parental education: Children without academic achievement data had parents with slightly lower attained education levels and were enrolled in schools with lower percentage of children with lower-educated parents than children with academic achievement data ($t(732) = -2.585, p = .010$, Cohen's $d = .191$; $t(1568) = -6.126, p < .001$, Cohen's $d = .318$, respectively).

School-level academic achievement: Children without academic achievement data were enrolled in schools with lower average academic achievement scores than children with academic achievement data ($t(992) = -3.441, p < .001$, Cohen's $d = .223$).

2. Comparison between included and excluded children in the final sample based on the preregistered inclusion criteria (*N* = 679)

The final sample of the present study was based on the preregistered inclusion criteria and included 679 participants. The remaining participants were excluded from the final sample.

Comparison regarding age and gender between exluded and included children in the final sample:

Age: In fourth grade, the excluded children were on average older (M_{years} = 10.09, SD_{years} = .50) than included children data (M_{years}= 9.96, SD_{years} = .40) in fourth grade, $t(1473) = 4.94$, $p < .001$, Cohen's $d = .258$. Furthermore, they were also slightly older in grades five and six

($t(1462) = 6.131$, $p <.001$, Cohen's $d = .300$; $t(1332) = 5.712$, $p < .001$, Cohen's $d = .322$, respectively).

Gender: There were no differences between excluded children and included children with respect to gender distribution, χ^2 (1) = 3.22, $p = .07$.

<u>Comparisons regarding academic self-concept (ASC) between exluded and included children in the final sample:</u>

Excluded and included children reported similar ASC levels in fourth, fifth and sixth grades ($t(1455) = -.745$, $p = .456$, Cohen's $d = .039$; $t(1449) = -.988$, $p = .323$, Cohen's $d = .052$; $t(1421) = -.947$, $p = .344$, Cohen's $d = .050$, respectively).

<u>Comparison regarding parental education between exluded and included children in the final sample:</u>

Household-level parental education: Excluded children with parental education data ($N = 55$) had parents with lower education levels compared to included children ($N = 679$), $t(732) = -4.179$, $p < .001$, Cohen's $d = .586$.

School-level parental education: Excluded children were enrolled in schools with higher percentage of children with lower-educated parents than included children ($t(1568) = 2.830$, $p = .005$, Cohen's $d = .144$)

<u>Comparisons regarding academic achievement between exluded and included children in the final sample:</u>

Child-level academic achievement: Excluded children had lower child-level academic achievement scores than included children ($t(559) = -4.770$, $p < .001$, Cohen's $d = .392$).

School-level academic achievement: Excluded children were in schools with lower average achievement scores than included children ($t(992) = -14.472$, $p < .001$, Cohen's $d = .918$).

<u>Comparisons between children with ($N = 340$) and without academic achievement data ($N = 339$) in the final sample ($N = 679$) and study variables:</u>

Age: Children without academic achievement data and children with academic achievement data were similar in their age across all grades (*ps* > .05). In grade six, children without academic achievement data were on average 11.94 years old ($SD = .50$) whereas and children with academic achievement data were on average 11.96 years old ($SD = .56$).

Gender: There were no differences in gender distribution between participants with academic achievement data and participants without academic achievement data (χ^2 (1) = 2.05, p = .156).

Academic self-concept (ASC): Compared to children without academic achievement data, children with academic achievement data reported slightly higher ASC levels in fourth and sixth grades but not in fifth grade ($t(661) = 3.164$, $p < .001$, Cohen's $d = .246$; $t(649) = 2.102$, $p = .036$, Cohen's $d = .165$; $t(660) = 1.125$, $p = .225$, Cohen's $d = .094$, respectively).

Household-level parental education: There were no significant differences in parental education levels between children with and without academic achievement data in the final sample ($t(677) = -1.489$, $p = .137$, Cohen's $d = .114$).

School-level parental education: Children without academic achievement data were enrolled in schools with lower percentage of children with lower-educated parents than children with academic achievement data ($t(677) = -2.842$, $p = .005$, Cohen's $d = .218$).

School-level academic achievement: Children without academic achievement data were enrolled in schools with higher average academic achievement scores than children with academic achievement data ($t(491) = 3.788$, $p < .001$, Cohen's $d = .368$).

Supplementary Note 2

Sensitivity Tests

Sensitivity tests were conducted to ensure the robustness of our results. The sensitivity tests were performed by using three models: 1) multiple imputation in Mplus with 25 imputed datasets, 2) single-level models, 3) mediation model based on a subsample of the final sample (those with complete achievement data, N =339). Single-level models were run both on 25 imputed datasets and on the final sample. Final sample (N = 679) refers to the sample based on preregistered inclusion criteria (see methods).

1. Multiple Imputation for Addressing Missing Data

To address the large proportions of missing data, we used multiple imputation techniques to impute the missing values of household- and school-level parental education data, child- and school-level academic achievement data and academic self-concept data. This means that we did not exclude children with two years of missing ASC data, with missing household- and school-level parental education and academic achievement data. In this larger sample, we had

1617 participants. We ran all the models with 25 imputed datasets. Below we present the results of these analyses with imputed datasets and compare them to the results of the model based on the final sample presented in the main manuscript.

Main effect and cross-level interaction model results:

The results of the main effect and cross-level interaction models are presented in Supplementary Table 1. The results at the household level lead to similar conclusions as the results reported in the main manuscript. Results showed significant associations between lower parental education and lower ASC levels in grades four, five and six. The results at the school level differed from the results presented in the main manuscript based on the final sample. That is, the results of the sensitivity analyses with multiple imputation did not show a significant association between school-level parental education and school-level ASC. Similarly, the results did not show that school-level parental education moderated the association between household-level parental education and child level-ASC.

Mediation model results:

The results of the mediation models are presented in Supplementary Table 2. Results from this model led to the same conclusions as the results presented in the main manuscript. At the household level, results showed a significant indirect effect, suggesting that lower household-level parental education was indirectly associated with lower ASC levels through lower levels of academic achievement (AA). At the school level, results showed that school-level AA did not mediate the association between school-level parental education and school-level ASC. There was a significant association between lower school-level parental education and lower school-level AA but the association between school-level AA and school-level ASC was not significant.

2. Single-level Models

Single-level models did not have a multi-level structure and used child-level ASC intercept parameter as the only outcome variable (rather than *both* child- and school-level ASC intercept parameters). In addition to the predictors at the child/household level, this model also included school-level variables (school-level parental education and school-level academic achievement) to predict child-level ASC. This approach eliminated the issue with the small number of clusters and was used to test the robustness of our results. The tests on single-level models were conducted using the larger sample (N = 1617) with imputed 25

datasets and the final sample ($N = 679$) used in the main MS, based on the preregistered inclusion criteria. The results from the single-level models generally lead to qualitatively similar conclusions as the conclusions reported in the main manuscript. Please see Supplementary Tables 3 - 6.

3. Mediation model based on a subsample with complete academic achievement data

Mediation model based on a subsample of the final sample included children with complete achievement data, resulting in 339 children and 10 schools. Due to the small number of clusters and a saddlepoint error in the multi-level model, a single-level model was performed to test the mediation model for this subsample. Similar to the results of the main model based on the final sample reported in the main manuscript and the results of the abovementioned sensitivity tests, the results showed significant indirect effect at the household level but not at the school level (See Supplementary Table 7). This suggests that child-level academic achievement was a significant mediator of the association between household-level parental education and child-level ASC. It should however be noted that the results obtained from this subsample of 339 participants may not be representative of the larger sample due to the differences between children with and without academic achievement data.

Supplementary Table 1

Results from multi-level main effect model and cross-level interaction model with imputed datasets (multiple imputation) (*N* = 1617)

	Academic Self-Concept Intercept (Grade 4 - Grade 6)				
**Main Effect Model**	*β*	*B*	*S.E.*	*p*	CI (95%)
**Within *(Child/household level)***					
	Child-level Academic Self-Concept				
Gender	.255	.111	.033	.001*	.047, .175
Household-level Parental Education	-.124	-.047	.018	.009*	-.081, -.012
**Between *(School level)***					
	School-level Academic Self-Concept				
School-level Parental Education	.225	.001	.003	.657	-.004, .007
**Cross-level Interaction Model**					
	Child-level Academic Self-Concept				
Intercept random intercept	-	-.042	.019	.030*	-.080, -.004
Household- x School-level Parental Education	-	.001	.002	.501	-.002, .004

Note. Household-level parental education scores were reverse coded so that higher scores indicate lower household-level parental education. Random intercept: the association between household-level parental education and child-level ASC. School-level parental education scores could range from 0% -100%, with higher percentage scores indicating lower school-level parental education. Note that the effect of school-level parental education represents the effect at 1% change in school-level parental education. Standardized regression coefficients are not available in MPLUS when testing cross-level interactions with the MLR estimator.

Supplementary Table 2

Results from the multi-level mediation model and mediation and interaction model with imputed datasets (multiple imputation) (*N* = 1617)

	Academic Achievement (Grade 6)					Academic Self-Concept (Grade 6)				
**Mediation Model**	*β*	*B*	*S.E.*	*p*	CI (95%)	*β*	*B*	*S.E.*	*p*	CI (95%)
**Within *(Child/household level)***	Child-level Academic Achievement					Child-level Academic Self-Concept				
Gender	-.006	-.057	.708	.936	-1.444, 1.331	.254	.110	.032	.001*	.048, .173
Household-level Parental Education	-.306	-2.345	.369	< .001*	-3.069, -1.622	.036	.013	.014	.355	-.015, .041
Child-level Academic Achievement	-	-	-	-	-	.508	.025	.002	< .001*	.021, .028
**Between *(School level)***	School-level Academic Achievement					School-level Academic Self-Concept				
School-level Parental Education	-.582	-.105	.028	< .001*	-.159, -.051	.391	.002	.003	.465	-.004, .009
School-level Academic Achievement	-	-	-	-	-	.364	.013	.014	.369	-.015, .040
						Child-level Academic Self-Concept				
Indirect Effect (mediation)	-	-	-	-	-	-	-.058	.010	< .001*	-.078, -.038
**Mediation and Interaction Model**										
Household- x School-level Parental Education	-	-	-	-	-	-	.001	.001	.496	-.002, .004
Indirect Effect (mediation)	-	-	-	-	-	-	-.058	.010	< .001*	-.078, -.038

Note. Household-level parental education scores were reverse coded so that higher scores indicate lower household-level parental education. School-level parental education scores could range from 0% -100%, with higher percentage scores indicating lower school-level parental education. Note that the effect of school-level parental education represents the effect at 1% change in school-level parental education. Standardized regression coefficients are not available in MPLUS when testing cross-level interactions with the MLR estimator.

Supplementary Table 3

Results from the single-level main effect model and interaction model based on the final sample (*N* = 679)

	Child-level Academic Self-Concept Intercept (Grade 4 - Grade 6)				
	β	*B*	*S.E.*	*p*	CI (95%)
**Main Effect Model**					
Gender	.250	.107	.035	.003*	.037, .176
Household-level Parental Education	-.124	-.041	.015	.005*	-.070, -.013
School-level Parental Education	.141	.012	.003	< .001*	.005, .019
**Interaction Model**					
Household- x School-level Parental Education	.135	.002	.001	.037*	.000, .004

Note. Household-level parental education scores were reverse coded so that higher scores indicate lower household-level parental education. School-level parental education scores could range from 0% -100%, with higher percentage scores indicating lower school-level parental education. Note that the effect of school-level parental education represents the effect at 1% change in school-level parental education.

Supplementary Table 4

Results from the single-level mediation model and mediation and interaction model based on the final sample (*N* = 679)

	Academic Achievement (Grade 6)					Academic Self-Concept (Grade 6)				
	β	*B*	*S.E.*	*p*	CI (95%)	*β*	*B*	*S.E.*	*p*	CI (95%)
**Mediation Model**	Child-level Academic Achievement					Child-level Academic Self-Concept				
Gender	.009	.076	.832	.928	-1.555, 1.706	.243	.104	.035	.003*	.035., .172
Household-level Parental Education	-.219	-1.408	.386	< .001*	-2.163, -.652	-.010	-.003	.015	.835	-.033. .027
Child-level Academic Achievement	-	-	-	-	-	.477	.025	.003	< .001*	.019, .030
	School-level Academic Achievement									
School-level Parental Education	-.284	-.084	.008	< .001*	-.099, -.069	.108	.009	.003	.005*	.003, .016
School-level Academic Achievement	-	-	-	-	-	-.006	-.002	.014	.895	-.029, .026
Indirect Effect (mediation)	-	-	-	-	-	-	-.035	.011	.001*	-.056, -.013
**Mediation and Interaction Model**										
Household- x School-level Parental Education	-	-	-	-	-	.153	.003	.001	.008*	.001, .004
Indirect Effect (mediation)	-	-	-	-	-	-	-.034	.011	.002*	-.055, -.013

Note. Household-level parental education scores were reverse coded so that higher scores indicate lower household-level parental education. Random intercept: the association between household-level parental education and ASC levels in sixth grade. School-level parental education scores could range from 0% -100%, with higher percentage scores indicating lower school-level parental education. Note that the effect of school-level parental education represents the effect at 1% change in school-level parental education. Standardized regression coefficients are not available in MPLUS when testing cross-level interactions with the MLR estimator.

Supplementary Table 5

Results from the single-level main effect model and interaction model with imputed datasets (*N* = 1617)

	Child-level Academic Self-Concept Intercept (Grade 4 - Grade 6)				
	β	*B*	*S.E.*	*p*	CI (95%)
**Main Effect Model**					
Gender	.236	.105	.025	<.001*	.056, .154
Household-level Parental Education	-.130	-.041	.015	.005*	-.069, -.012
School-level Parental Education	.071	.005	.002	.041*	.000, .010
**Interaction Model**					
Household- x School-level Parental Education	.168	.002	.002	.201	-.001, .006

Note. Household-level parental education scores were reverse coded so that higher scores indicate lower household-level parental education. School-level parental education scores could range from 0% -100%, with higher percentage scores indicating lower school-level parental education. Note that the effect of school-level parental education represents the effect at 1% change in school-level parental education.

Supplementary Table 6

Results from the single-level mediation model and mediation and interaction model with imputed datasets (multiple imputation) (*N* = 1617)

	Academic Achievement (Grade 6)					Academic Self-Concept (Grade 6)				
	β	*B*	*S.E.*	*p*	CI (95%)	*β*	*B*	*S.E.*	*p*	CI (95%)
**Mediation Model**	Child-level Academic Achievement					Child-level Academic Self-Concept				
Gender	.010	.102	.764	.894	-1.395, 1.598	.233	.105	.027	< .001*	.052, .158
Household-level Parental Education	-.345	-2.284	.333	< .001*	-2.936, -1.632	.051	.016	.020	.426	-.024, .056
Child-level Academic Achievement	-	-	-	-	-	.523	.025	.002	< .001*	.021, .029
	School-level Academic Achievement									
School-level Parental Education	-.333	-.090	.007	< .001*	-.103, -.076	.026	.002	.003	.520	-.004, .008
School-level Academic Achievement	-	-	-	-	-	-.093	-.025	.014	.064	-.051, .002
Indirect Effect (mediation)	-	-	-	-	-	-	-.057	.011	< .001*	-.079, -.035
**Mediation and Interaction Model**										
Household- x School-level Parental Education	-	-	-	-	-	.164	.002	.002	.151	-.001, .006
Indirect Effect (mediation)	-	-	-	-	-	-	-.056	.010	< .001*	-.075, -.037

Note. Household-level parental education scores were reverse coded so that higher scores indicate lower household-level parental education. Random intercept: the association between household-level parental education and ASC levels in sixth grade. School-level parental education scores could range from 0% -100%, with higher percentage scores indicating lower school-level parental education. Note that the effect of school-level parental education represents the effect at 1% change in school-level parental education. Standardized regression coefficients are not available in MPLUS when testing cross-level interactions with the MLR estimator.

Supplementary Table 7

Results from the single-level mediation model based on a subsample with complete achievement data (*N* = 339)

	**Academic Achievement (Grade 6)**					**Academic Self-Concept (Grade 6)**				
	β	*B*	*S.E.*	*p*	CI (95%)	*β*	*B*	*S.E.*	*p*	CI (95%)
	Child-level Academic Achievement					Child-level Academic Self-Concept				
**Main Effect Model**										
Gender	-	-	-	-	-	.282	.124	.051	.015*	.024, .223
Household-level Parental Education	-	-	-	-	-	.036	.013	.021	.525	-.027, .053
School-level Parental Education	-	-	-	-	-	.194	.014	.004	< .001*	.006, .022
**Mediation Model**										
Gender	.023	.189	.874	.829	-1.525, 1.902	.260	.113	.046	.014*	.023, .204
Household-level Parental Education	-.147	-.978	.406	.016*	-1.773, -.183	.125	.045	.019	.021*	.007, .082
Child-level Academic Achievement	-	-	-	-	-	.470	.025	.003	< .001*	.019, .031
	School-level Academic Achievement									
School-level Parental Education	-.241	-.056	.014	< .001*	-.083, -.029	.134	.010	.003	.004*	.003, .017
School-level Academic Achievement	-	-	-	-	-	-.027	-.008	.018	.630	-.043, .026
Indirect Effect (mediation)	-	-	-	-	-	-	-.025	.011	.024*	-.046, -.003

Note. Household-level parental education scores were reverse coded so that higher scores indicate lower household-level parental education. School-level parental education scores could range from 0% -100%, with higher percentage scores indicating lower school-level parental education. Note that the effect of school-level parental education represents the effect at 1% change in school-level parental education.

Supplementary Note 3

After running the mediation model based on the final sample, we also ran a mediation model where we included cross-level interactions (see the Mediation and Cross-level Interaction Model under Table 3 in the main manuscript). In this model, similar to the results from RQ1b, we also found a significant cross-level interaction between household- and school-level parental education on child-level ASC in sixth grade. Probing the interaction effects using 0.5 SD above and below the mean of school-level parental education showed that the association between household-level parental education and ASC levels was not significant in both higher (B = -.008, *S.E.* = .025, p = .746, CI [-.058, .042]) and lower parental education (B = .017, *S.E.* = .020, p = .385, CI [-.021, .055]) schools. The association between household-level parental education and child-level ASC only started to significantly differ when school-level parental education percentage was 1.3 SD above the mean of school-level parental education. This suggests that when schools have 21% or more children with lower-educated parents (i.e., lower parental education schools), children of lower educated parents reported higher ASC than children of higher-educated parents (B = .037, *S.E.* = .019, p = .050, CI [.000, .074]). There were no differences in child-level ASC when schools had less than 21% of children from lower-educated households.
